# Model-based optimization of combination protocols for irradiation-insensitive cancers

**DOI:** 10.1038/s41598-020-69380-6

**Published:** 2020-07-28

**Authors:** Beata Hat, Joanna Jaruszewicz-Błońska, Tomasz Lipniacki

**Affiliations:** Institute of Fundamental Technological Research, Polish Academy of Sciences, Warsaw, Poland

**Keywords:** Cancer, Systems biology

## Abstract

Alternations in the p53 regulatory network may render cancer cells resistant to the radiation-induced apoptosis. In this theoretical study we search for the best protocols combining targeted therapy with radiation to treat cancers with wild-type p53, but having downregulated expression of PTEN or overexpression of Wip1 resulting in resistance to radiation monotherapy. Instead of using the maximum tolerated dose paradigm, we exploit stochastic computational model of the p53 regulatory network to calculate apoptotic fractions for both normal and cancer cells. We consider combination protocols, with irradiations repeated every 12, 18, 24, or 36 h to find that timing between Mdm2 inhibitor delivery and irradiation significantly influences the apoptotic cell fractions. We assume that uptake of the inhibitor is higher by cancer than by normal cells and that cancer cells receive higher irradiation doses from intersecting beams. These two assumptions were found necessary for the existence of protocols inducing massive apoptosis in cancer cells without killing large fraction of normal cells neighboring tumor. The best found protocols have irradiations repeated every 24 or 36 h with two inhibitor doses per irradiation cycle, and allow to induce apoptosis in more than 95% of cancer cells, killing less than 10% of normal cells.

## Introduction

The tumor suppressor p53 plays a major role in cell cycle suppression and apoptosis initiation in response to DNA damage^1^. It suppresses propagation of genetic mutations and prevents cancer initiation and development^2^. Unsurprisingly, the p53 gene is the most commonly mutated gene found in human cancers^3^. Nevertheless, more than half of all cancers retain wild-type (WT) p53^3^. In these cancers, p53 signaling is suppressed or deregulated due to various alternations in the p53 pathway components. These include overexpression of a p53 negative regulator, Mdm2^4^, overexpression of phosphatase Wip1 (dephosphorylating both a DNA damage sensor, ATM^5^, and p53^6^), or downregulation of expression of a pro-apoptotic phosphatase PTEN^7^.

PTEN is a tumor-suppressing phosphatase that catalyzes dephosphorylation of PIP3 to PIP2, resulting in inhibition of the AKT pathway regulating cell growth and survival. PTEN is one of the most commonly lost tumor suppressors in human cancers^8–10^. Deletions, mutations, transcriptional silencing of the PTEN gene, or protein instability were reported in endometrial carcinoma, glioblastomas, skin, breast, prostate, colorectal, gastric cancers; reviewed by Bermúdez Brito et al*.*^7^. Loss of one PTEN gene allele dramatically increases breast^11^ and prostate^12^ cancer progression. In glioblastoma^13^ and several breast cancers, despite intact PTEN gene locus, PTEN level is reduced due to post-transcriptional regulation by microRNAs^14^. Even 20% reduction of PTEN level results in the increased levels of cyclin D1 and phosphorylated AKT^15,16^ and itself induces various tumors in mice^15,17^. Cancer cells with reduced PTEN level have enhanced proliferation rate and increased resistance to UV radiation^15^.

Wip1 overexpression abrogates apoptosis by decreasing the tumor-suppressive activity of p53, promotes cell proliferation and is associated with poor survival prognosis^6,18,19^. *PPM1D*, coding for Wip1, is amplified in many human tumors, including breast cancer^20,21^, neuroblastoma^22^, ovarian clear cell carcinomas^23^, medulloblastomas^24^, gliomas^25^, pancreatic adenocarcinomas^26^, gastric carcinomas^27^, colorectal cancer^28^.

Our previous modeling study^29^ showed that responsiveness of cancer cell lines to irradiation can be diminished by decreased expression of PTEN or increased expression of Wip1. This is in line with experimental studies^30–34^. In nasopharyngeal carcinoma cells, PTEN inhibition increases resistance to irradiation^30^. Overexpression of Wip1 reduces radiation-induced apoptosis in prostate cancer^31^ and cisplatin-induced apoptosis in ovarian clear cell adenocarcinomas^32^ and in intestinal crypts and testes (normal tissue) of transgenic (pUbC-Wip1) mouse^33^ with WT p53. In turn, reduced Wip1 expression increases sensitivity of MCF-7 cell line to doxorubicin^34^ as well as of the osteosarcoma cell line U2OS and the colon cancer cell line HCT116 expressing WT p53 to cisplatin^33^.

The p53 interaction with Mdm2 is one of the most intensively studied therapeutic targets. A number of small-molecule inhibitors have been developed to target this interaction by (1) inhibiting Mdm2 stability^35^, (2) inhibiting the E3 ligase activity of Mdm2^36,37^, or (3) inhibiting the p53-Mdm2 binding^38–40^. Intensively studied are inhibitors from the nutlin (*cis*-imidazolines) family, small-molecules that prevent p53–Mdm2 interaction by binding to the hydrophobic p53-binding pocket of Mdm2^41^. The preclinical studies showed that nutlin-3 increases p53 concentration and induces apoptosis or senescence in numerous, mostly WT p53 cell lines of osteosarcoma^38,42^, lung cancers^43^, prostate cancers^44^, colon cancer^42^, melanoma^45^, ovarian cancer^46^, neuroblastoma^47^ and renal cancer^48^. RG7112 from the nutlin family induces apoptosis and increases p53 phosphorylation in ovarian clear cell carcinoma^49^ and leukemia (I phase of clinical trial)^50^.

Importantly, in the context of our study, nutlin-3 sensitizes lung cancer^43^, laryngeal carcinoma^51^, esophageal squamous^52^ and prostate^44,53^ cancer cells, retaining WT p53, to radiation. Nutlin-3 also enhances effectiveness of DNA-damaging chemotherapy in MCF7 breast cancer^54,55^, neuroblastoma^56^, B cell chronic lymphocytic leukemia^57^ and sarcoma^58^ cells. TDP665759, a benzodiazepinedione inhibitor of the Mdm2:p53 complex, increases cancer cells sensitivity to doxorubicin in A375 melanoma cell culture and xenograft^59^.

A number of models were proposed to optimize scheduling of cancer therapy protocols^60^. Most of mathematical models used for optimization operate at the cell population level^61,62^, using e.g. birth–death branching processes^63^, or Gompertzian tumor growth dynamics^64^ without taking into account molecular details of the p53 pathway. Population-level modeling allowed to apply the optimal control theory to search for optimal protocols for long term mono and combination therapies^65–67^. Mathematical models provided support for metronomic therapies, in which drug is used at sufficiently low dose, such that patient recovery breaks, in which remaining cancer cells may grow exponentially, are not needed^68^. Combination of continuous low and intermitted high dosing may delay development of cancer resistance arising due to repeated phases of selection and clonal expansion^69^. Optimization of therapy protocols based on analysis of a drug influence on molecular pathway dynamics are only emerging^70–74^.

**Figure 1:**
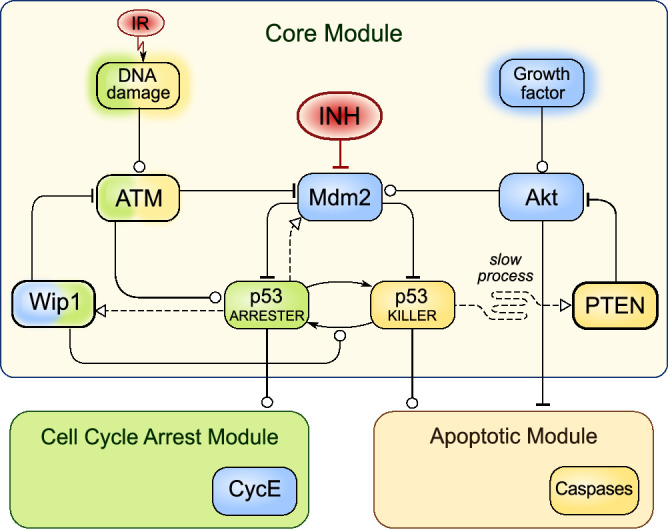
Simplified scheme of the model (based on Hat et al.^29^). Arrow-headed dashed lines—positive transcriptional regulation; arrow-headed solid lines—protein transformation, circle-headed solid lines—positive influence or activation; hammer-headed solid lines—inhibitory regulation. Pro-survival and cell cycle promoting proteins are shown within blue boxes, pro-apoptotic proteins are within yellow boxes, proteins involved in cell cycle arrest are within green boxes. Two inputs/treatments (red circles) are considered: Mdm2 inhibitor (INH) and γ irradiation (IR). The detailed schematic of the model is given in Supplementary Fig. S1 (in the Supplementary Text S1).

In this study we perform a protocol optimization based on the molecular p53 pathway model^29^, which allows us to take into account specific alternations in this pathway. The p53 model exhibits biphasic dynamics^75,76^, in which an oscillatory response to the DNA damage is either followed by a return to the resting state after a transient cell cycle arrest (for small DNA damage) or by apoptosis following cell cycle arrest in the case when the DNA repair lasts too long. The early version of the model^76^ was applied to study nutlin monotherapy, by Puszyński et al., who explored effects of the stochastic gene regulation and found that dose-splitting may be ineffective at low doses and effective at high doses^71^. Here, we apply the Hat et al. model (see Fig. 1 for a simplified cartoon and Supplementary Fig. S1 for a detailed scheme) that captures antagonistic actions of Wip1 and PTEN^29^ and consider combination therapies involving γ irradiation and inhibitor of Mdm2–p53 interaction (such as nutlin). We will focus on cancer cell lines with WT p53 and elevated expression of Wip1 (termed for brevity Wip1-cancer cells), or with reduced expression of PTEN (referred to as PTEN-cancer cells). In our previous study we demonstrated that such cells are not sensitive to radiation monotherapy^29^.

In standard (not metronomic) protocols chemotherapeutic agents or irradiation are given at their maximum tolerated doses^77^. Our results suggest, however, that especially for combination therapies the effect of treatment on normal cells does not solely depend on the cumulative drug and irradiation doses but also on the treatment schedule. We therefore propose a different approach, in which we search for protocols that are most harmful to cancer cells and least damaging to normal cells. We will assume that cancer cells receive higher irradiation dose than normal cells, as it is in the case of treatments with intersecting beams that form radiation isocenter. We will also assume that the applied Mdm2 inhibitor has higher uptake by cancer cells, and estimate minimal specificity ratio, cancer-to-normal cells, for the inhibitor to make the combination therapy effective and safe for normal cells. We will use two types of simulations: deterministic (solving ODE approximation of the model) and stochastic (simulating Markov process). Deterministic approximation allows for fast initial selection of the most promising protocols. Based on stochastic simulations, for the selected protocols we will calculate the fraction of apoptotic cells and choose the acceptable protocols for which apoptotic fraction for cancer cells exceeds 95% and for normal cells that receive fractional irradiation doses remains below 10%.

## Methods

### Model and numerical simulations

Analysis of therapeutic protocols is based on computational p53 model developed in Hat et al.^29^, supplemented by equations describing Mdm2 inhibitor liberation to blood, its elimination as well as its association with (and dissociation from) Mdm2. The detailed schematic of the model is shown in Supplementary Fig. S1 and model parameters are provided in the Supplementary Text S1. The deterministic analysis is based on ordinary differential equations accounting for mass action or Michaelis–Menten kinetics of the concentrations of mRNAs and proteins considered in the model. In the stochastic approach the model dynamics is described by the time-continuous Markov process and simulated using the Gillespie algorithm^78^. Stochastic kinetic Monte Carlo simulations were performed in BioNetGen^79^. To accelerate simulations of the model the scaling method was applied^80^. Fractions of apoptotic cells for each protocol were calculated based on 1,000 stochastic simulations. Matlab and BioNetGen scripts with model code are provided in ZIP-archived directories Supplementary Code S1 and Code S2.

### Cell types and therapeutic protocols

We consider three cell types: normal cells modeled with the nominal parameter values, cancer cells with either fivefold increased Wip1 transcription rate (termed Wip1-cancer cells), or cells with fivefold reduced PTEN transcription rate (termed PTEN-cancer cells). Simulated therapeutic protocols consist of 12, 18, 24, or 36-h long cycles (cycle is defined as a time between subsequent irradiations). In the case of combination therapy, Mdm2 inhibitor is administered once per 12- and 18-h cycle or twice per 24- and 36-h cycle. Preselection of optimal combination therapy protocols is based on deterministic simulations. In these simulations we analyze protocols consisting of either 14 cycles of length 24 or 36 h, or 28 cycles of length 12 or 18 h. Final selection and verification of optimal protocols is based on stochastic simulations, in which apoptotic cell fractions are determined. These simulations are performed for preselected protocols consisting of 7 cycles of length 24 or 36 h.

The inhibitor dose (INH) is given in IC_50_ units per single administration or per day, where IC_50_ is the half maximal inhibitory concentration. In these units the equilibrium fraction of inhibitor-bounded Mdm2 is F = INH_B_/(1 + INH_B_), where INH_B_ is the inhibitor level in blood, lower than the administered dose due to drug elimination. In the case of oral administration, inhibitor liberation rate coefficient (to blood) is assumed d_r1_ = 1/h, while inhibitor elimination rate coefficient is d_r2_ = 0.25/h. Thus, in the case of drip drug delivery the equilibrium INH_B_ is reached in about 4 h, while in the case of oral delivery it oscillates during treatment, having maximum about 2 h after drug delivery as observed experimentally in Zhang et al.^75^. The processes of inhibitor translocation to the cell and Mdm2 binding are lumped together. We assume that cancer cells have either equal or higher absorption rate than normal cells. In the latter case, for the sake of simplicity, we assume that for normal cells the inhibitor–Mdm2 association rate constant k_a_ is lower (either 3 or 10 times) than for cancer cells. This assumption is equivalent to the assumption that the effective inhibitor dose (in IC_50_ units) for normal cells is lower than for cancer cells. Inhibitor dose is always given with respect to cancer cells.

### Protocol selection and optimization method

The protocol selection was performed independently for Wip1-cancer and PTEN-cancer cells. We analyzed 10 combination protocols with oral inhibitor delivery (see “Results”). The analysis was performed under the assumption that cancer and normal cells have equal inhibitor absorption rates or that cancer cells have 3 or 10 times higher absorption rate. The analysis of the last case is documented in “Results”, while the analogous analysis in the first and in the second case is documented in the Supplementary Text S1 (see Supplementary Fig. S2). In each case for 10 combination protocols, using deterministic approximation of the model, we determined critical irradiation dose inducing apoptosis (IR_crit_) for normal and cancer cells as a function of inhibitor dose. Next, we calculate IR_crit_ ratio of normal to Wip1-cancer and PTEN-cancer cells (i.e. IR_crit_normal_/IR_crit_PTEN_cancer_ and IR_crit_normal_/IR_crit_Wip1_cancer_, respectively) as a function of inhibitor dose. Typically, this ratio has a single maximum suggesting the optimal inhibitor dose for each protocol.

Next, for all 10 protocols we performed further stochastic model analysis considering inhibitor doses at maximum of IR_crit_ ratio. For each selected protocol we performed 1,000 stochastic simulations of cancer cells and of normal cells applying to the latter threefold lower irradiation dose; this reflects the assumption that when beams intersect at tumor, neighboring cells receive lower dose of irradiation than cancer cells. The search for optimal dose IR_opt_ (i.e. such for which the fraction of apoptotic cancer cells exceeds 95% and for normal cells is less than 10%) was performed in the range $\left[0.8, 1.5\right]$ × IR_crit_cancer_.

## Results

### Radiation monotherapy protocols

**Figure 2:**
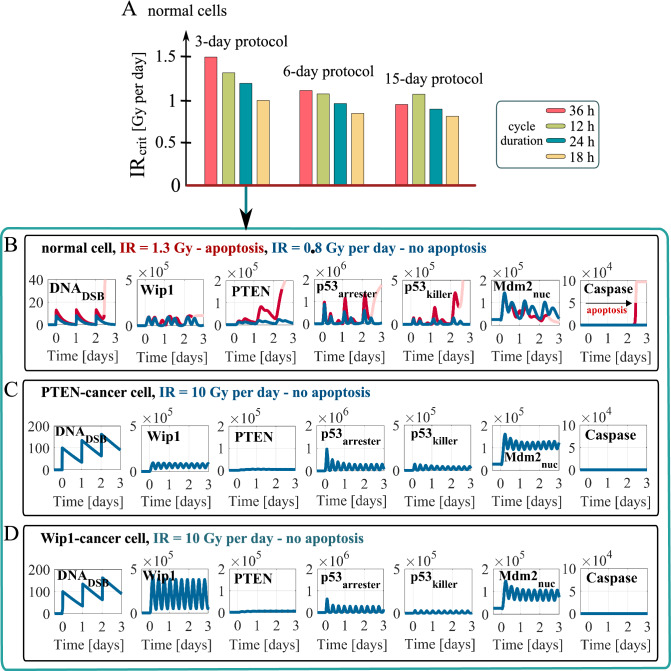
Responses of normal, PTEN-cancer and Wip1-cancer cells to different irradiation protocols. (**A**) Critical irradiation dose for normal cells for 3-, 6- and 15-day protocols with irradiation cycles lasting either 12, 18, 24 or 36 h. (**B,C,D**) deterministic simulation trajectories in response to the 3-day protocol with 24-h irradiation cycle (with dose per day as specified) for normal, PTEN-cancer and Wip1-cancer cells. The faded line section in (B) visualizes the trajectory after the initiation of apoptosis.

Using deterministic simulations we calculate the minimal irradiation dose per day (denoted by IR_crit_), at which normal cells (i.e. with nominal transcription rate coefficients of Wip1 and PTEN: s_1_ = 0.1 mRNA/s, s_2_ = 0.03 mRNA/s, respectively), undergo apoptosis in response to a given protocol (Fig. 2A). Considering 3-, 6- and 15-day protocols with irradiation cycles lasting 12, 18, 24 or 36 h, we found that IR_crit_ decreases with the length of protocol (mostly between 3- and 6-day protocols), and that the most cytotoxic protocols (i.e. with the lowest IR_crit_) are these with cycle length equal 18 h. For this cycle length IR_crit_ is about 0.8 Gy per day for 6- and 15-day treatments. Ranking of protocols is the same for the three considered treatment lengths, with one exception that for 3- and 6-day therapies the protocol with 12 h-cycle is better than the one with 36 h-cycle, in contrast to 15-day therapy.

In Fig. 2B,C,D we show 3-day long trajectories of the key p53 system components in response to 24-h irradiation cycle protocol, most commonly used in radiotherapy. We consider normal cells (Fig. 2B), PTEN-cancer cells (with s_2_ = 0.006 mRNA/s, i.e., fivefold lower than the nominal value, Fig. 2C) and Wip1-cancer cells (with s_1_ = 0.5 mRNA/s, i.e. fivefold higher than the nominal value, Fig. 2D).

In the case of normal cells subcritical repeated dose of 0.8 Gy leads to forced periodic oscillations (of period 24 h) of p53_arrester_ (controlling expression of Mdm2 and Wip1) and p53_killer_ (controlling expression of PTEN), while the supercritical repeated dose of 1.3 Gy induces apoptosis in the third cycle. In contrast, we found that PTEN-cancer and Wip1-cancer cells are completely resistant to DNA-damage-induced apoptosis, as they withstand persistent DNA damage induced by 10 Gy repeated dose (Fig. 2C,D). These cells exhibit DNA damage driven oscillations with period equal about 7 h (much shorter than irradiation cycle), however with the amplitude of p53_killer_ not exceeding the level required to induce apoptosis. Resistance to DNA damage-induced apoptosis, and semi-periodic oscillations lasting at least 3 days, were observed by Geva-Zatorsky et al. in single MCF7 cells that are known to have downregulated PTEN expression^81^.

### Combination therapy protocols

#### Impact of the Mdm2 inhibitor administration on the critical irradiation dose

We showed that PTEN-cancer and Wip1-cancer cells are resistant to DNA-damage-induced apoptosis (Fig. 2C,D), which implies that for these cells radiation monotherapy cannot be effective. For that reason we propose to combine radiotherapy with Mdm2 inhibitor delivery. First, let us notice, that within frames of the considered model, Mdm2 inhibitor alone is not able to induce apoptosis. By suppressing Mdm2 ubiquitinase activity, inhibitor leads to an increase of p53 level, but without DNA damage signal, p53 remains unphosphorylated at Ser 15 and thus may not serve as a transcription factor. In fact, Vassiliev et al. demonstrated that nutlin-1 (in contrast to DNA damaging agents) does not lead to p53 phosphorylation at Ser 15^41^. However, when administered at high dose, either nutlin-1 or nutlin-3 lead to the increase of Mdm2 and p21 levels, which suggests that unphosphorylated p53 retains some transcriptional activity, and may suppress cell viability^82,83^. Because of the critical role of Ser 15 phosphorylation in busting p53 activity^84^, we expect that Mdm2 inhibitor administered alone requires much higher dose, than when it is administered as a part of irradiation protocol that leads to rapid p53 phosphorylation at Ser 15. Consequently, we restrict to the model in which DNA-damage induced phosphorylation of p53 is necessary for its activation and induction of cell apoptosis.

**Figure 3:**
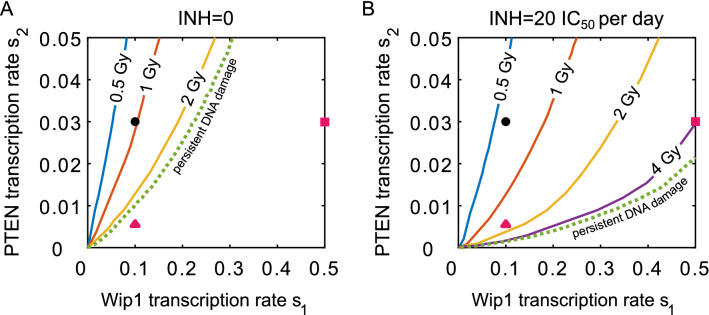
Critical irradiation doses as a function of Wip1 (s_1_) and PTEN (s_2_) transcription rate coefficients without and with Mdm2 inhibitor. Color lines show the critical irradiation doses in the (s_1_, s_2_)-parameter plane, for 6-day monotherapy (**A**) and combination therapy (**B**) protocols. For each dose, the line separates the apoptotic region (above the line) and the survival region (below the line). Black dot corresponds to the nominal values of s_1_ and s_2_ (normal cells), red square corresponds to Wip1-cancer cells, and red triangle corresponds to PTEN-cancer cells. Irradiation cycle is 24 h, while the inhibitor (in **B**) is administrated twice per cycle, 6 and 18 h after irradiation, each time at the dose equal 10 IC_50_.

In Fig. 3 we consider a 24-h cycle protocol and show that inhibitor administration reduces critical irradiation dose for all values of s_1_ and s_2_. The inhibitor is given twice per cycle (Fig. 3B): 6 and 18 h after irradiation, each time at the same dose equal 10 IC_50_, where IC_50_ is the half maximal inhibitory concentration (see “Methods”). Figure 3A confirms that Wip1-cancer cells (with fivefold increase of s_1_ from the nominal value, red square), or PTEN-cancer cells (with fivefold decrease of s_2_, red triangle) are resistant to persistent DNA damage. These cells, however, become apoptotic when the combination therapy is applied (Fig. 3B). For monotherapy (Fig. 3A) red square and triangle corresponding to Wip1-cancer and PTEN-cancer cells are outside the apoptotic region (for persistent DNA damage), while in the case of combination therapy (Fig. 3B) Wip1-cancer and PTEN-cancer cells are within the apoptotic region.

#### Normal and cancer cells in response to combination therapy

**Figure 4:**
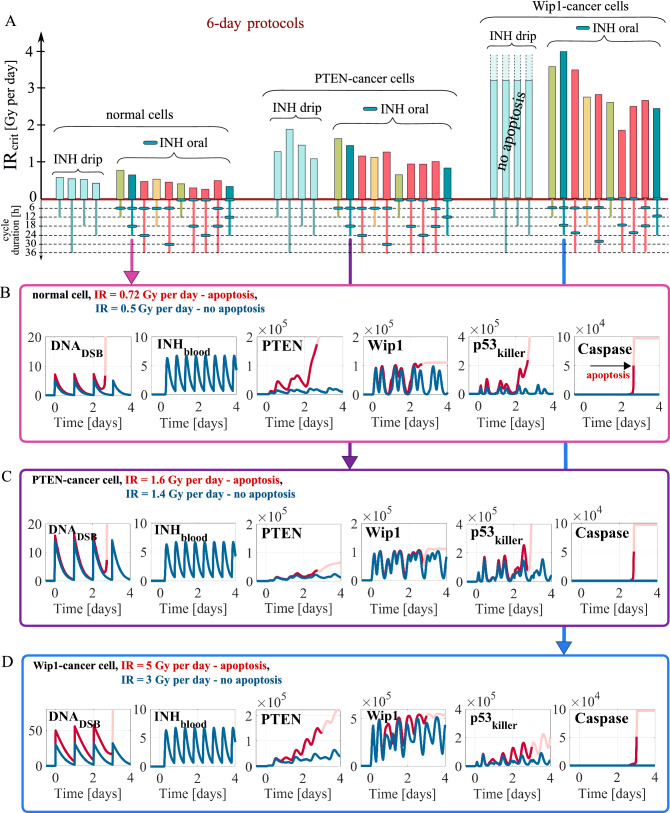
Critical irradiation doses and simulation trajectories for normal and cancer cells in response to combination therapies. (**A**) Critical irradiation doses for 6-day protocols after drip and oral inhibitor administration estimated in deterministic model simulations. Different bar colors correspond to different cycle durations, 12 (green), 18 (yellow), 24 (blue) and 36 h (red). Cells are irradiated at the beginning of each cycle, while the inhibitor is administrated at a day dose of 20 IC_50_ at time points indicated by blue ovals (in the case of oral administration). (**B,C,D**) Deterministic simulation trajectories for normal, PTEN-cancer and Wip1-cancer cells in response to two irradiation doses, one above (red) and the other below (blue) IR_crit_, for the protocol as in Fig. 2. The faded line segments visualize trajectories after the initiation of apoptosis.

In previous subsection we demonstrated that the combination protocol (in contrast to monotherapy) can trigger apoptosis both in Wip1-cancer and PTEN-cancer cells. Consequently, for normal and cancer cells we analyze 10 different 6-day protocols with 12, 18, 24 or 36-h cycles (Fig. 4). The inhibitor is given either once during 12- and 18-h cycles, or twice during 24 and 36-h cycles, or continuously in the case of drip administration with the inhibitor dose equal 20 IC_50_ per day.

We may notice that the critical irradiation dose strongly depends on protocol (Fig. 4A). The general tendency is that for protocols, in which inhibitor is administered simultaneously with irradiation, the critical dose is lower with respect to the remaining protocols. The protocols with continuous drip inhibitor administration have higher values of IR_crit_ than the best protocols with oral inhibitor administration. Although drip administration allows to keep the inhibitor in blood at a constant level, the same dose of inhibitor per day administered orally results in temporally higher inhibitor concentrations. Timing between irradiation and inhibitor delivery is important since in response to the DNA damage p53_killer_ level exhibits oscillations of a 7-h period, with an amplitude dependent on an actual inhibitor level in blood, which in the case of oral administration is oscillatory (Figs. 4B,C,D). Oscillations of p53_killer_ are further enhanced by the positive feedback mediated by PTEN and lead to apoptosis.

Importantly, comparing normal and cancer cells, we may notice that critical irradiation doses for normal cells are the lowest, while for Wip1-cancer cells are the highest. For Wip1-cancer cells, the drip inhibitor administration (at daily dose of 20 IC_50_) does not lead to apoptosis even in the case of persistent DNA damage (caused by high repeated doses of irradiation). In further analysis we restrict to protocols with oral inhibitor administration. For all 10 considered protocols inhibitor, when delivered orally, is able to sensitize PTEN-cancer and Wip1-cancer cells to irradiation. However, due to the protective effect of PTEN downregulation or Wip1 overexpression, cancer cells receiving the same dose of inhibitor require higher irradiation dose than normal cells. This suggests that inhibitor to be safe and effective must have higher uptake by cancer than by normal cells. Only such inhibitor would allow to reduce IR_crit_ for cancer cells to the value lower than or comparable to IR_crit_ for normal cells. In further analysis, when searching for optimal protocols, we will focus on the case in which uptake of the inhibitor is 10 or 3 times higher by cancer than by normal cells. The case of the equal inhibitor absorption is analyzed in Supplementary Fig. S2.

In Figs. 4B, 4C and 4D we show deterministic simulation trajectories for normal, PTEN-cancer and Wip1-cancer cells in response to two irradiation doses, one above IR_crit_, the other below IR_crit_. As the example protocol we choose the same one as in Fig. 3. Later on we will demonstrate that this protocol is the optimal among 24-h cycle protocols. First, let us notice that due to inhibitor elimination its level oscillates and remains below the single administration dose 10 IC_50_.

Comparing trajectories for PTEN-cancer cells for monotherapy (Fig. 2C) with these for combination therapy (Fig. 4C) we may notice that Mdm2 inhibitor administration leads to an increase of p53_killer_ oscillation amplitude. This in turn increases accumulation of PTEN (which is in positive feedback loop with p53_killer_), and allows for triggering apoptosis. Attenuation of Mdm2-p53 negative feedback loop by inhibitor administration allows for induction of apoptosis in PTEN-cancer cells at irradiation dose equal 1.6 Gy per day (Fig. 4C), while without inhibitor these cells remain resistant to repeated dose equal 10 Gy per day that induces persistent DNA damage (Fig. 2C). A higher irradiation dose of 3.98 is needed to trigger apoptosis in Wip1-cancer cells (Fig. 4D). In these cells Wip1 exhibits high amplitude oscillations controlling the level of p53_killer_ by dephosphorylating it to the p53_arrester_ form (see Fig. 1). As a consequence, p53_killer_ exhibits oscillatory behavior even in the apoptotic cell trajectory in contrast to normal and PTEN-cancer cells when apoptosis is preceded by sharp increase of p53_killer_ level (compare Fig. 4D with Fig. 4B,C).

**Figure 5:**
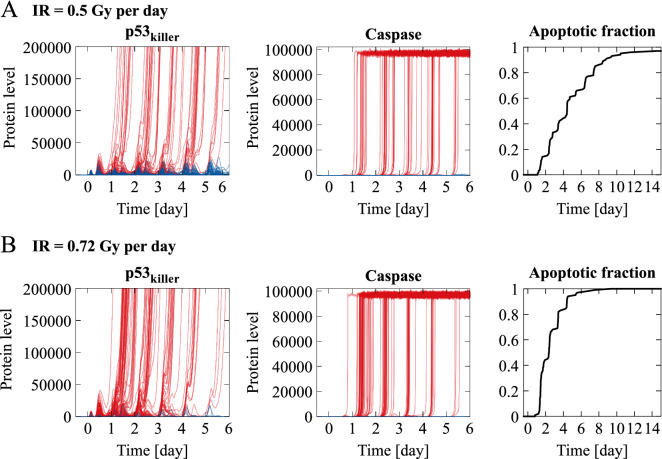
Stochastic simulation trajectories and apoptotic cell fraction for normal cells in response to combination therapy. The same protocol and the same irradiation doses, above IR_crit_ (**A**) and below IR_crit_ (**B**), as in Fig. 4B are used. Red lines represent p53_killer_ and Caspase levels of cells committing to apoptosis within first 6 days, blue lines represent surviving cells trajectories.

In Fig. 5 we show stochastic simulation trajectories for normal cells in response to the same protocol as in Fig. 4B and with the same two irradiation doses below and above the IR_crit_ equal to 0.61 Gy per day for that protocol. As expected, irradiation dose 0.72 Gy per day exceeding IR_crit_ results in massive apoptosis (97 out of 100 cells in first 6 days), but even for the dose 0.5 Gy per day, below IR_crit_, a fraction of cells each day commits to apoptosis, as a consequence of stochasticity. As a result, the apoptotic fraction exceeds 60% after 6 days and 90% after 15 days. Discrepancy between deterministic and stochastic behavior suggests that conclusions regarding efficiency of a given protocol must be verified in stochastic simulations, that reflect heterogeneous responses across cell population.

### Plausible and optimal protocols

As a plausible we consider protocol with a low apoptotic fraction of normal cells, not exceeding 10%, and high apoptotic fraction of cancer cells, at least 95%. As an optimal we will consider the plausible protocol with the lowest possible dose of inhibitor. We select plausible protocols for the combination therapies under the following assumptions:

1. Normal cells in the vicinity of tumor receive a threefold lower irradiation dose than cancer cells (this can be achieved by irradiating tumor with intersecting beams).
2. Inhibitor uptake by cancer cells is equal to that of normal cells or is either 3- or tenfold higher. The analyses of these three inhibitor specificity ratios are performed independently.
3. Normal and cancer cells retain WT p53 and all other components of the considered p53 regulatory pathway.
4. PTEN-cancer cells have a fivefold lower expression of PTEN, while Wip1-cancer cells have a fivefold higher expression of Wip1.

**Figure 6:**
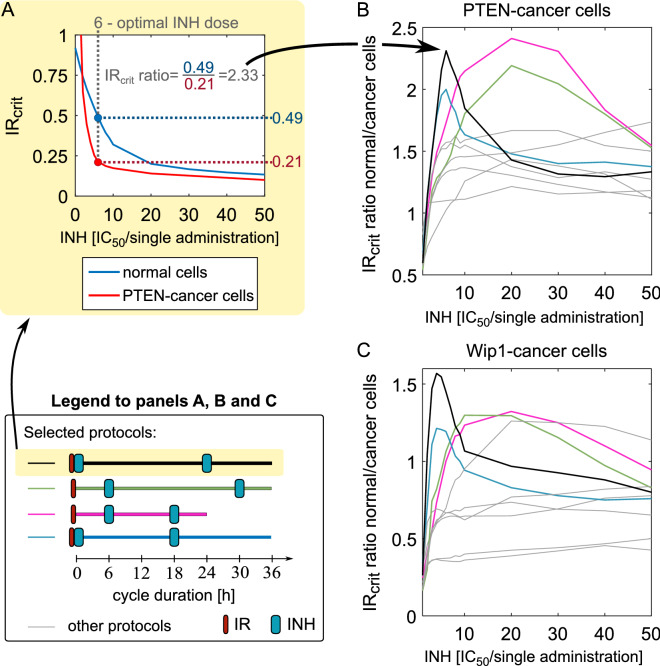
Relative resistance to irradiation: normal versus PTEN-cancer and Wip1-cancer cells. The inhibitor dose is given in IC_50_ units per single administration with respect to cancer cells for which tenfold higher (with respect to normal cells) specificity is assumed. (**A**) Panel explains how the IR_crit_ ratio for cancer-to-normal cells was calculated. (**B**) and (**C**) Relative resistance to irradiation based on the deterministic model simulations. Color lines correspond to the four protocols (see Legend) that based on stochastic simulations were found plausible for both PTEN- and Wip1-cancer cells. Grey lines correspond to the other analyzed protocols (shown in Fig. 4A).

The search for plausible protocols is performed in two steps; the first one involves deterministic model simulation of all 10 protocols with oral inhibitor delivery shown in Fig. 4A, the second one involves stochastic simulations (see “Methods” for details). Based on the deterministic simulations we calculate IR_crit_ ratio: normal to cancer cells. In Fig. 6 we show the case in which inhibitor uptake is tenfold higher by the cancer than by the normal cells, while in Supplementary Fig. S2 we show cases in which uptake by the cancer cells is either threefold higher or equal to that of the normal cells. Intuitively, the higher is the IR_crit_ ratio the more resistant to radiation are the normal cells with respect to the cancer cells. For each protocol IR_crit_ ratio is a function of inhibitor dose, and attains maximum for some value of inhibitor dose. Since both PTEN- and Wip1-cancer cells are resistant to radiation alone, IR_crit_ ratio equals zero for zero inhibitor dose. As shown in Fig. 6B,C, IR_crit_ ratio is higher for PTEN-cancer cells than for Wip1-cancer cells. All considered protocols for PTEN-cancer cells have the IR_crit_ ratio greater than 1.0 for some range of inhibitor dose, while for Wip1-cancer cells for half of protocols maximum IR_crit_ ratio is below 1.0. In the case in which uptake by cancer cells is only threefold higher than that of normal cells, IR_crit_ ratio reaches 0.7 for Wip1-cancer cells (for the two most promising protocols) and 1.0 for PTEN-cancer cells, also for two protocols, see Suplementary Fig. S2. Under assumption that inhibitor uptake is equal for the normal and the cancer cells, IR_crit_ ratio remains below 0.4 for Wip1-cancer cells and below 0.7 for PTEN-cancer cells.

Next, for all 10 protocols we conduct further stochastic model analysis performing simulations with inhibitor doses at which IR_crit_ ratio attains maximum. Based on the stochastic simulations we found that in the case when the inhibitor specificity ratio (cancer vs normal cells) equals 10, all 10 protocols are plausible for PTEN-cancer cells for some irradiation dose. In the case of Wip1-cancer cells only four out of 10 protocols (shown in Fig. 6C by color lines) were found plausible. Unfortunately, only one of them is the 24-h cycle protocol feasible to use in real treatment, while the remaining three have 36-h cycles. The four protocols found plausible for Wip1-cancer cells are also highlighted in color in Fig. 6B for PTEN-cancer cells, and not surprisingly they exhibit high IR_crit_ ratios. It is worth noticing that the protocols in which inhibitor is delivered simultaneously with irradiation, have maximum IR_crit_ ratio at low inhibitor dose.

**Figure 7:**
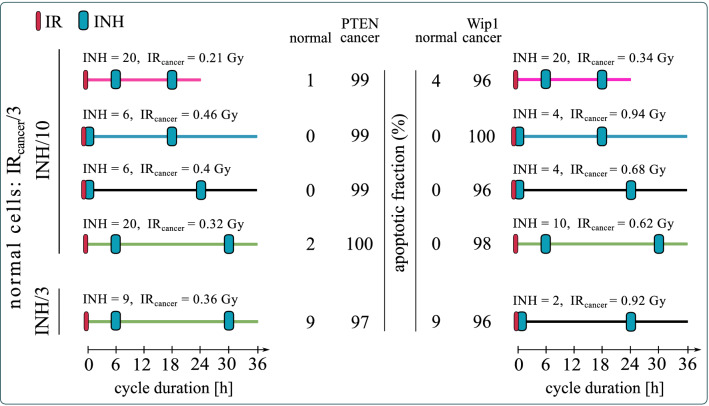
Apoptotic fractions of normal, PTEN-cancer and Wip1-cancer cells for the four plausible protocols. All protocols lasted 7 cycles. Cancer cells receive irradiation dose IR_cancer_ (as given); normal cells receive threefold lower irradiation dose and have 10 or threefold lower inhibitor uptake (as given) than cancer cells. In the latter case there exists only one plausible protocol (i.e. such for which the apoptotic fraction of normal cells remains below 10% and apoptotic fraction of cancer cells exceeds 95%).

In Fig. 7 we show the apoptotic cell fractions for normal and cancer cells obtained in numerical simulations of the four protocols that were found plausible for both PTEN- and Wip1-cancer cells under the assumption that inhibitor specificity ratio (cancer vs normal cells) equals 10. For each protocol the inhibitor dose is selected (based on analysis in Fig. 6) such that IR_crit_ ratio attains maximum. The search for plausible irradiation dose was performed in the range $\left[0.8, 1.5\right]$ × IR_crit_cancer_. For each protocol we show the inhibitor and irradiation dose for which the fraction of apoptotic cells was calculated. In the case when inhibitor specificity ratio equals 3, we found only one plausible protocol for PTEN-cancer and (different) for Wip1-cancer cells. These two protocols are within two pairs of protocols with the highests IR_crit_ ratio for PTEN-cancer and for Wip1-cancer cells (Supplementary Fig. S2A—green line and S2B—black line). Finally, as expected, for the case in which the inhibitor uptake is equal for the normal and for the cancer cells we have not found any plausible protocol.

**Figure 8:**
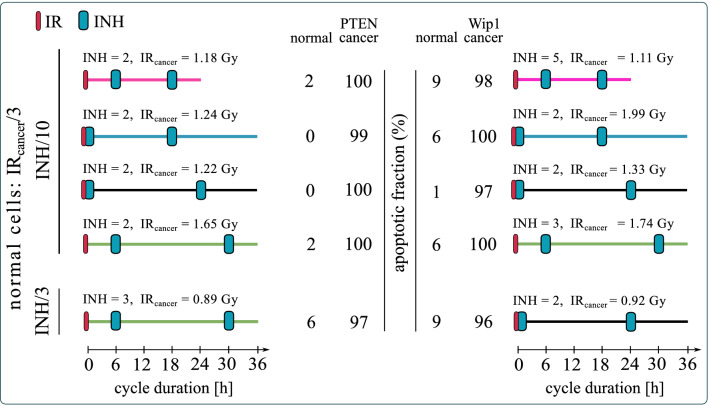
Apoptotic fractions of normal, PTEN-cancer and Wip1-cancer cells for the optimal protocols. Cancer cells receive irradiation dose IR_cancer_ (as given); normal cells receive threefold lower irradiation dose and have 10 or threefold lower inhibitor uptake (as given) than cancer cells. The same protocols as in Fig. 7 are considered but with the minimized inhibitor dose under the condition that the apoptotic fraction of normal cells remains below 10% and apoptotic fraction of cancer cells exceeds 95%.

Finally, we ask the question whether the inhibitor dose can be reduced, still allowing to find such an irradiation dose that the apoptotic fraction of normal cells (in the vicinity of tumor) will remain below 10% and apoptotic fraction of cancer cells will exceed 95%. Although, in our model, the inhibitor alone is not leading to apoptosis, it is obvious that all inhibitors have side effects, and thus their dose should be as small as possible. As shown in Fig. 8, for all selected protocols the inhibitor dose can be reduced, and both for PTEN- and Wip1-cancer cells there are protocols for which the inhibitor dose equals 2 IC_50_. Overall, based on Fig. 8, the most promising protocols for PTEN-cancer are: (1) a feasible 24-h cycle protocol with inhibitor delivery 6 and 18 h after irradiation. This protocol requires 2 IC_50_ dose of inhibitor which is assumed to be tenfold more specific to cancer than to normal cells (so the effective dose for normal cells is 0.2 IC_50_); (2) 36-h cycle protocol with inhibitor delivery 6, and 30 h after irradiation. This protocol requires a less selective inhibitor, only threefold more specific to cancer than to normal cells at the dose of 3 IC_50_ (corresponding to 1 IC_50_ for normal cells). For Wip-1 the 36-h cycle protocols require a lower inhibitor dose than the one with 24-h cycle. The most promising one has drug delivery simultaneously, and 24 h after irradiation. This protocol requires inhibitor that is only threefold more specific to cancer than to normal cells at the dose of 2 IC_50_ (corresponding to 0.67 IC_50_ for normal cells).

## Discussion

### Primum non nocere

The goal of each cancer therapy is to eliminate cancer cells (or at least reduce their number), without severely endangering patient life. Thus, to perform protocol optimization one needs to define constrains that reflect the necessity to protect the patient. The most common approach refers to the maximum tolerated dose paradigm^77^ and thus implicitly bases on the assumption that toxicity of a given therapy with respect to normal, non-cancer cells depends solely on the total cumulated dose and/or maximum daily dose. There is however no reason to expect that cancer cells’ fate depends on the protocol schedule while fate of non-cancer cells does not. In contrast, we observe that the impact of a given protocol on cancer as well as normal cells depends not only on the irradiation and inhibitor doses, but also on the specific scheduling of the treatment.

We have thus proposed a new methodology of investigating the therapeutic protocols in which we use the mathematical modeling and analyze an impact of a given protocol on cancer cells, as well as on non-cancer cells. In order to distinguish between two types of cells we made use of the p53 network model^29^ within which we may account for cells having different expression of cell fate-controlling phosphatases PTEN and Wip1. We assume also that the non-cancer cells adjacent to the tumor receive a lower irradiation dose and a lower effective dose of the Mdm2 inhibitor. The first effect can be obtained by the use of intersecting beams, the latter follows from the assumed slower uptake of inhibitor by normal cells. The majority of normal cells, that are distant from tumor, receive only Mdm2 inhibitor, which alone, within frames of our p53 network model^29^, is not lethal. This simplification is justified for low inhibitor doses of order of IC_50_, and thus in final protocol optimization step we searched for protocols with the lowest inhibitor dose. We have focused on Mdm2 inhibitors, because these inhibitors are widely studied in clinical trials^36–40^, but the same analysis can be performed within frames of the computational model, for other inhibitors including inhibitors of Wip1^54,55^.

We performed our search for optimal protocols in two steps: first, we used deterministic ODE approximation of the model to find protocols for which the ratio of critical irradiation doses: normal-to-cancer cells is the highest; then, we performed massive stochastic simulations of the model to find irradiation doses for which the apoptotic fraction in cancer cells exceeds 95%, while in normal neighboring cells remains below 10%. The stochastic step of analysis is important since, as demonstrated, even the protocols with subcritical irradiation dose can induce massive apoptosis over time, due to repeated irradiations.

In stochastic simulations, we accounted only for the intrinsic noise, i.e., noise associated with small number of reacting molecules (such as gene copies, or DNA strand breaks). An important role is played, however, by the extrinsic noise, associated with the pretreatment heterogeneity of population and resulting in different sensitivity to the inhibitor or radiation. Extrinsic noise may substantially modify probability densities of the fate-controlling phosphatases PTEN and Wip1^85^ and thus influence apoptotic fractions of both normal and cancer cells. One of the major problems associated with high-dose therapies is the selection of the most resistant cancer clones, which after a treatment may undergo exponential expansion. Metronomic therapies or treatments combing short high dose and long low dose drug periods were proposed to tackle this problem^68,69^.

Overexpression of Wip1 or reduced expression of PTEN are a signatures of numerous cancers of reduced sensitivity to radiotherapy^30–34^. This motivates the search for combination protocols targeting the p53 inhibitor, Mdm2, and in this way sensitizing cancer cells to radiotherapy. We considered cancer cells having either a fivefold overexpression of Wip1 or a fivefold decreased expression of PTEN, which, within frames of the model, makes them insensitive to the radiotherapy alone. Of note, radiotherapy alone may induce apoptosis in normal cells (that is, in cells with the nominal expression of PTEN and Wip1), while combination therapies using an Mdm2 inhibitor may also induce apoptosis of cancer cells. Assuming that cancer cells receive a threefold higher irradiation dose and have a tenfold higher uptake of inhibitor, we found four protocols (applicable to PTEN- and Wip1-cancers) for which the apoptotic fraction for normal neighboring cells is less than 10%, while the apoptotic fraction of cancer cells exceeds 95%. The most potentially applicable protocol is the one in which irradiations are administered every 24 h, with inhibitor delivered 6 and 18 h post each irradiation. Unfortunately, the remaining protocols have 36-h cycles (irradiations are administered every 36 h). Since the tenfold inhibitor specificity difference between normal and cancer cells can be difficult to achieve, we considered also the case in which cancer cells have only threefold higher inhibitor uptake than normal cells. Under this assumption we were able to find one protocol for PTEN-cancer and one (different) for Wip1-cancer satisfying the desired criteria of normal *versus* cancer cells apoptosis. Both protocols have 36-h cycles. Interestingly, for these two protocols, it is possible to use the low inhibitor doses of respectively, 3 IC_50_ and 2 IC_50_, thus not exceeding 1 IC_50_ with respect to normal cells, which should assure small toxicity for normal non-irradiated cells that receive inhibitor only. Under the assumption that the inhibitor uptake is equal for the normal and for the cancer cells we have not found any plausible protocol.

We have focused on protocols combining irradiation and Mdm2 inhibitor, as a potential treatment for tumors with modified expression of PTEN and Wip1. Similar analysis, however, can be performed for therapies involving DNA-reactive agents that are a natural choice in the case of metastatic cancers for which irradiation may not be effective^86^. In such a case, in addition to the differences in expression of proteins specific for a considered cancer, one should account for the different uptake rates of specific inhibitor and DNA-reactive agent by normal and cancer cells.

Our results suggest that the combination therapy protocols can be promising for tumors characterized by a reduced expression of PTEN or increased expression of Wip1, that are insensitive to radiation monotherapy. It is naive to expect however, that the found therapeutic protocols can be universal for all tumors with modified expression of these two phosphatases. In the case of specific tumor cell line, one should first adjust the model parameters, then follow the described procedure to find optimal protocols to be validated in experimental tests. Impressive example of parameter estimation enabling prediction of responses of various cancer cell lines was presented by Frohlich et al.^72^, who integrated RAS/MAPK and AKT signaling pathways into model accounting for more than 100 genes and 36 activating mutations yielding a total of 1,228 molecular species. However, in order to apply the proposed protocol selection procedure one should also characterize responses of normal cells from healthy tissues, that may have different sensitivity to irradiation and inhibitor.

In conclusion, our analysis indicates that (1) an outcome of a given protocol strongly depends on the specific timing between irradiations and inhibitor deliveries. In the case of tumors characterized by reduced expression of PTEN or increased expression of Wip1 the protocols with irradiations every 36 h seems more promising than these with irradiations every 24 h (or less than 24 h). (2) The maximum tolerated dose paradigm should be treated with caution since the apoptotic fraction in cancer cells as well as normal cells strongly depends on the irradiation and inhibitor delivery schedule. (3) Instead of using the maximum tolerated dose paradigm one can consider simultaneously cancer and normal cells, and based on calculated apoptotic fractions in these two populations select the optimal protocols. Formal therapy design is intended to guide experiments, and of course, requires experimental validation of candidate protocols.

## Supplementary information

Supplementary information.

S1 Code.

S2 Code.

## Data Availability

All data generated or analyzed during this study are included in this published article and its Supplementary Information files.
