# Supplementary material for: Model-based optimization of combination protocols for irradiation-insensitive cancers
Source: Sci Rep. 2020 Jul 28;10:12652. doi: 10.1038/s41598-020-69380-6 (PMC7387345; doi:10.1038/s41598-020-69380-6)
Supplement: Supplementary file 1 — Supplementary information. [file 41598_2020_69380_MOESM1_ESM.pdf]

Supporting Information
S1-S2 Figures and S1-S3 Tables

featuring the article

Model-based optimization of combination protocols for irradiation-insensitive cancers

by Beata Hat[1], Joanna Jaruszewicz-Błońska[1], and Tomasz Lipniacki[1]

[1] Institute of Fundamental Technological Research, Polish Academy of Sciences, Warsaw, Poland

Scientific Reports 2020

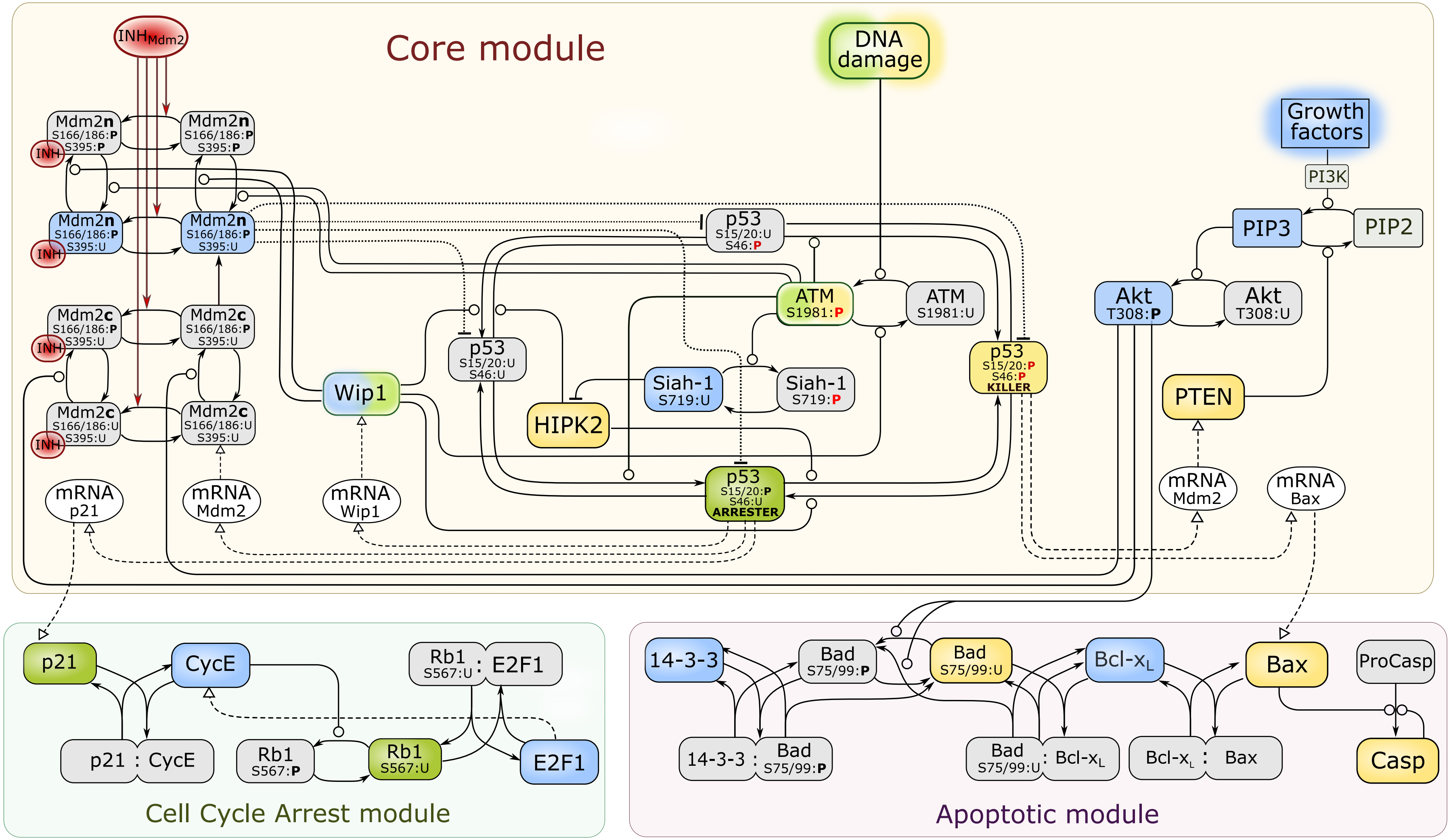

S1 Figure. Detailed representation of the full model based on [S1]. Arrow-headed dashed lines indicate transcriptional regulation, arrow-headed solid lines—protein transformation, circle-headed solid lines—positive influence, hammer-headed dotted lines—ubiquitination by Mdm2 leading to protein degradation. The subscripts n or c denote either nuclear or cytoplasmic localization of Mdm2. Bold 'P' and non-bold 'U' denote phosphorylated and unphosphorylated states of given residues, respectively. Mdm2 inhibitor is represented with red circle; pro-survival and cycle-promoting proteins are represented with blue boxes, pro-apoptotic proteins with yellow boxes, proteins promoting cell cycle arrest with green boxes, while the remaining proteins and protein complexes are left in grey boxes.

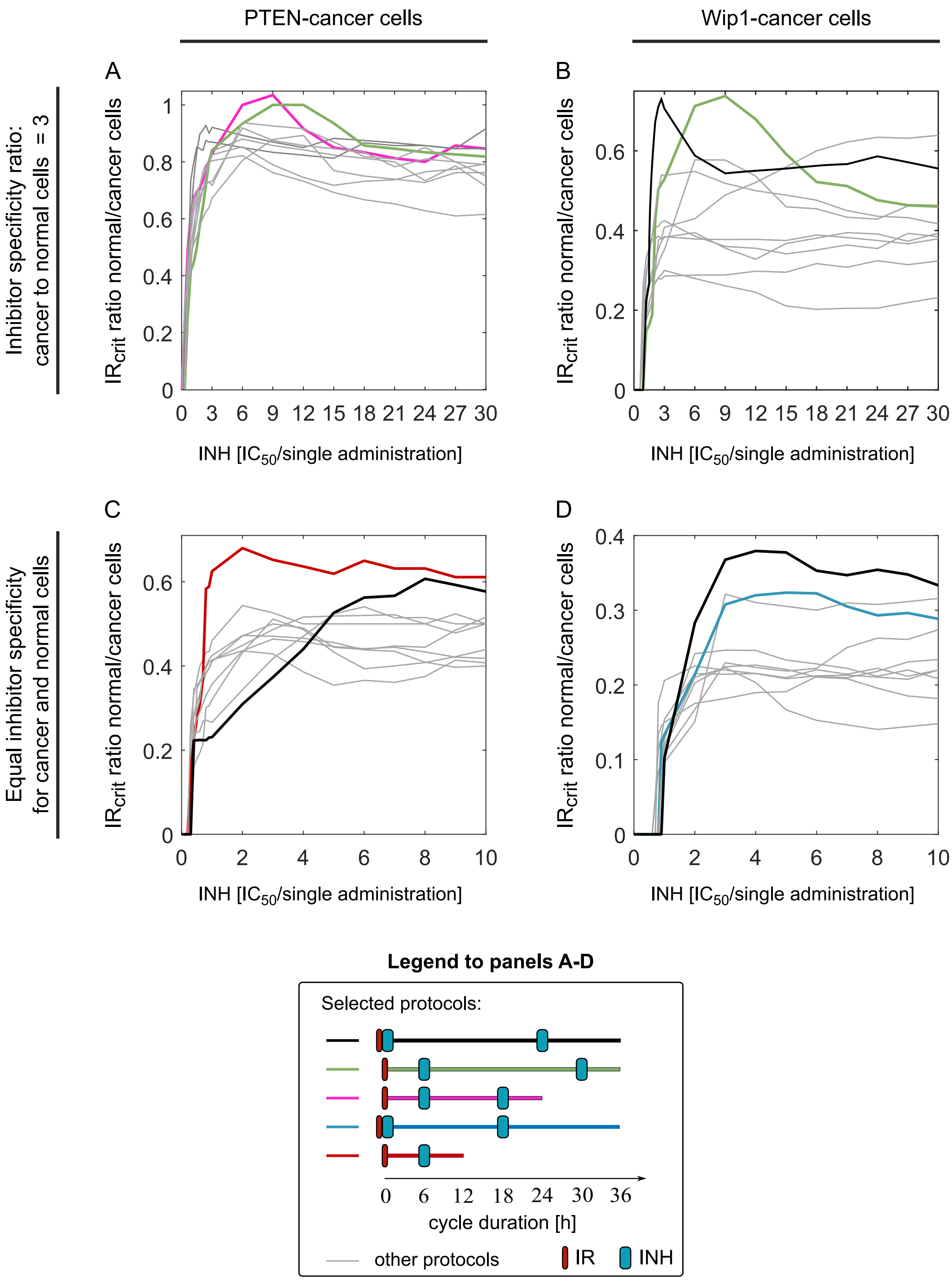

S2 Figure. Relative resistance to irradiation: normal versus PTEN-cancer (**A**, **C**) and Wip1-cancer (**B**, **D**) cells based on the deterministic model simulations. The inhibitor dose is given in IC_{50} units per single administration with respect to cancer cells for which 3-fold higher (**A-B**) or equal (**C-D**) specificity (with respect to normal cells) is assumed. In each panel two protocols with highest IR_{crit} ratio are marked with color lines (see Legend). Grey lines correspond to the other analyzed protocols (shown in Fig. 4A).

All parameters, excluding parameters governing inhibitor dynamics, come from our previous study [S1]. All reactants levels are given in molecules/cell; such representation enables stochastic simulations. Parameters governing inhibitor dynamics where chosen to reproduce experimental data shown in Zhang et al. [S2]. The maximal level of inhibitor in blood is achieved at approximately 2 hours after oral inhibitor delivery. After next 10 hours the inhibitor level decreases approximately ten-fold (see Fig 4). For the sake of simplicity processes of cellular import of inhibitor from blood and its binding with Mdm2 are lumped together into a single reaction with rate k_a. Analogously, inhibitor:Mdm2 complex dissociation is lumped with inhibitor export from cell to blood and described by a single reaction with rate k_d. Oral inhibitor delivery is instantaneous. Technical realization of such delivery is following: inhibitor administration time is set equal 10 seconds and the single dose, denoted by INH, is administered with rate INH/10 s^{-1}. In the case of drip inhibitor delivery, a daily dose INH is given with rate INH/24 h^{-1}. We assume that at any given time point the fraction of inhibitor molecules bound to Mdm2 is small; accordingly we neglect depletion of inhibitor due to Mdm2 binding.

Supporting Tables

S1 Table. Notation guide.

Symbol	Description
**Inhibitor dynamics**	
INH_{Mdm2_oral}	Mdm2 inhibitor administered orally
INH_{Mdm2_blood}	Mdm2 inhibitor level in blood
$Mdm2_{\mathrm{cyt\_0p}} : INH_{\mathrm{Mdm2blood}}$	complex of $\mathrm{INH_{Mdm2\_blood}}$ and cytoplasmic unphosphorylated Mdm2
$Mdm2_{\mathrm{cyt\_2p}} : INH_{\mathrm{Mdm2blood}}$	complex of $\mathrm{INH_{Mdm2\_blood}}$ and cyt. Mdm2 p'ylated at Ser166 and 186
$Mdm2_{\mathrm{nuc\_2p}} : INH_{\mathrm{Mdm2blood}}$	complex of $\mathrm{INH_{Mdm2\_blood}}$ and nuclear Mdm2 p'ylated at Ser166 and 186
$Mdm2_{\mathrm{nuc\_3p}} : INH_{\mathrm{Mdm2blood}}$	complex of $\mathrm{INH_{Mdm2\_blood}}$ and nuc. Mdm2 p'ylated at Ser166, 186 and 395
**Core module**	
DNA_{DSB}	DNA damage due to IR: double strand breaks (DSBs)
ATM_u	unphosphorylated kinase ATM
ATM_{p}	ATM phosphorylated at Ser1981 (upon DNA DSBs)
$Wip1_{\mathrm{gene\_a}}$, $Wip1_{\mathrm{gene\_i}}$	state of the Wip1 (PPM1D) gene: active(a)/inactive(i)
$Wip1_{\mathrm{mRNA}}$	Wip1 transcript
Wip1	phosphatase Wip1
$SIAH1_{\mathrm{u}}$	unphosphorylated SIAH1
$SIAH1_{\mathrm{p}}$	SIAH1 phosphorylated at Ser19
HIPK2	kinase HIPK2
$p53_{\mathrm{0p}}$	unphosphorylated p53
$p53_{\mathrm{ARRESTER}}$	p53 phosphorylated at Ser15, Ser20
$p53_{\mathrm{KILLER}}$	p53 phosphorylated at Ser15, Ser20 and additionally at Ser46
$p53_{\mathrm{s46}}$	p53 phosphorylated at Ser46 only
$Mdm2_{\mathrm{gene\_a}}$, $Mdm2_{\mathrm{gene\_i}}$	state of the Mdm2 gene: active(a)/inactive(i)
$Mdm2_{\mathrm{mRNA}}$	Mdm2 transcript
$Mdm2_{\mathrm{cyt\_0p}}$	cytoplasmic, unphosphorylated Mdm2
$Mdm2_{\mathrm{cyt\_2p}}$	cytoplasmic Mdm2 phosphorylated at Ser166 and Ser186
$Mdm2_{\mathrm{nuc\_2p}}$	nuclear Mdm2 phosphorylated at Ser166 and Ser186
$Mdm2_{\mathrm{nuc\_3p}}$	nuclear Mdm2 phosph. at Ser166, 186 and additionally at Ser395
PI3K	kinase PI3K
$PTEN_{\mathrm{gene\_a}}$, $PTEN_{\mathrm{gene\_i}}$	state of the PTEN gene: active(a)/inactive(i)
$PTEN_{\mathrm{mRNA}}$	PTEN transcript
PIP2	bi-phosphatidylinositol
PIP3	tri-phosphatidylinositol

Akt_u	unphosphorylated AKT
Akt_p	Akt phosphorylated at Thr308
**Apoptotic module**	
Bax_{gene_a}, Bax_{gene_i}	state of the Bax gene: active(a)/inactive(i)
Bax_{mRNA}	Bax transcript
Bax	unbound form of Bax
$Bclx_L$	unbound form of Bcl-x_L
$Bax : Bclx_L$	complex of Bax and Bcl-x_L
Bad_u	unbound, unphosphorylated Bad
Bad_p	Bad: unbound, phosphorylated at Ser75 and Ser99
$Bcl\text{-}x_L : Bad_u$	complex of Bcl-x_L and Bad_u
14-3-3	unbound adapter protein 14-3-3
Bad_p: *14-3-3*	complex of Bad_p and 14-3-3
proCasp	inactive caspase
Casp	active caspase
**Cell cycle arrest module**	
$p21_{gene\_a}$, $p21_{gene\_i}$	state of the p21 gene: active(a)/inactive(i)
$p21_{mRNA}$	p21 transcript
p21	unbound p21
CycE	unbound Cyclin E
p21: *CycE*	complex of p21 and Cyclin E
$Rb1_u$	Rb1: unbound, unphosphorylated at Ser780
$Rb1_p$	Rb1: unbound, phosphorylated at Ser780
$Rb1_u : E2F1$	complex of unphosphorylated Rb1 and E2F1

S2 Table. Model parameters.

Parameter	Symbol	Value	Remarks
Duration of the IR phase	IR_T	600 [s]	—
Inhibitor dose	INH_{dose}	as given	—
Irradiation dose	IR_{Gy}	as given	—
Number of DSBs per 1Gy of IR	DSB_{Gy}	10 [break/cell]	—
Maximal number of DSBs	DSB_{max}	10^6 [break/cell]	—
Number of repair complexes	DSB_{rep}	20 [mlc/cell]	—
Total amount of SIAH1	$SIAH1_{tot}$	10^5 [mlc/cell]	$SIAH1_{tot} = SIAH1_u + SIAH1_p$
Total amount of ATM	ATM_{tot}	10^5 [mlc/cell]	$ATM_{tot} = ATM_u + ATM_p$
Total amount of BclXL	$BclXL_{tot}$	10^5 [mlc/cell]	$Bclx_{L_{tot}} = Bclx_L + Bclx_L{:}Bad_u + Bclx_L{:}Bax$
Total amount of Bad	Bad_{tot}	6×10^4 [mlc/cell]	$Bad_{tot} = Bad_u + Bad_p + Bclx_L{:}Bad_u +$ $+Bad_p$: 14-3-3
Total amount of Rb1	$Rb1_{tot}$	3×10^5 [mlc/cell]	$Rb1_{tot} = Rb1_p + Rb1_u + Rb1_u{:}E2F1$

Total amount of E2F1	$E2F1_{tot}$	2×10^5 [mlc/cell]	$E2F1_{tot} = E2F1 + Rb1_u{:}E2F1$
Total amount of Akt	Akt_{tot}	10^5 [mlc/cell]	$Akt_{tot} = Akt_u + Akt_p$
Total amount of PIP3 and PIP2	PIP_{tot}	10^5 [mlc/cell]	$PIP_{tot} = PIP2 + PIP3$
Total amount of 14-3-3	$14\text{-}3\text{-}3_{tot}$	2×10^5 [mlc/cell]	$14\text{-}3\text{-}3_{tot} = 14\text{-}3\text{-}3 + Bad_p{:}\,14\text{-}3\text{-}3$

S3 Table. Reactions and reaction rate coefficients. All reactants levels are in molecules/cell.

Reaction	Rate	Coeff.	Value
**Inhibitor dynamics**			
$\varnothing \xrightarrow{INH} \boldsymbol{INH_{Mdm2\_oral}}$	$\frac{INH_{dose}}{duration}$	duration	10 s (oral single administration) 24×3600 s (drip administration)
$\boldsymbol{INH_{Mdm2\_oral}} \rightarrow \boldsymbol{INH_{Mdm2\_blood}}$	dr_1	dr_1	2.8×10^{-4}/s
$\boldsymbol{INH_{Mdm2\_blood}} \rightarrow \varnothing$	dr_2	dr_2	0.7×10^{-4}/s
$\boldsymbol{Mdm2_{cyt\_0p}} \xrightarrow{INH_{Mdm2\_blood}} \boldsymbol{Mdm2_{cyt\_0p}{:}INH_{Mdm2\_blood}}$	$k_a \cdot INH_{Mdm2\_blood}$	k_a	k_d
$\boldsymbol{Mdm2_{cyt\_2p}} \xrightarrow{INH_{Mdm2\_blood}} \boldsymbol{Mdm2_{cyt\_2p}{:}INH_{Mdm2\_blood}}$			
$\boldsymbol{Mdm2_{nuc\_2p}} \xrightarrow{INH_{Mdm2\_blood}} \boldsymbol{Mdm2_{nuc\_2p}{:}INH_{Mdm2\_blood}}$			
$\boldsymbol{Mdm2_{nuc\_3p}} \xrightarrow{INH_{Mdm2\_blood}} \boldsymbol{Mdm2_{nuc\_3p}{:}INH_{Mdm2\_blood}}$			
$\boldsymbol{Mdm2_{cyt\_0p}} \leftarrow \boldsymbol{Mdm2_{cyt\_0p}{:}INH_{Mdm2\_blood}}$	k_d	k_d	4.6×10^{-5}/s
$\boldsymbol{Mdm2_{cyt\_2p}} \leftarrow \boldsymbol{Mdm2_{cyt\_2p}{:}INH_{Mdm2\_blood}}$			
$\boldsymbol{Mdm2_{nuc\_2p}} \leftarrow \boldsymbol{Mdm2_{nuc\_2p}{:}INH_{Mdm2\_blood}}$			
$\boldsymbol{Mdm2_{nuc\_3p}} \leftarrow \boldsymbol{Mdm2_{nuc\_3p}{:}INH_{Mdm2\_blood}}$			
$\boldsymbol{Mdm2_{cyt\_2p}{:}INH_{Mdm2\_blood}} \rightarrow \boldsymbol{Mdm2_{nuc\_2p}{:}INH_{Mdm2\_blood}}$	i_1	i_1	10^{-3}/s
$\boldsymbol{Mdm2_{nuc\_2p}{:}INH_{Mdm2\_blood}} \xrightarrow{ATM_p} \boldsymbol{Mdm2_{nuc\_3p}{:}INH_{Mdm2\_blood}}$	$p_6 \cdot ATM_p$	p_6	10^{-8}/s
$\boldsymbol{Mdm2_{nuc\_2p}{:}INH_{Mdm2\_blood}} \xleftarrow{Wip1} \boldsymbol{Mdm2_{nuc\_3p}{:}INH_{Mdm2\_blood}}$	$d_6 \cdot Wip1$	d_6	10^{-10}/s

Core module			
$\varnothing \xrightarrow{IR} \boldsymbol{DNA_{DSB}}$	$h_1 \cdot \frac{DSB_{Gy} \cdot IR_{Gy}}{IR_T} \cdot (DSB_{\max} - DNA_{DSB})$	h_1<br>DSB_{Gy}<br>IR_T<br>$DSB_{\max}$<br>h_2	10^{-6}/s<br>10<br>10×60 s<br>10^6<br>10^{-13}/s
$\varnothing \xrightarrow{Casp} \boldsymbol{DNA_{\mathrm{DSB}}}$	$h_2 \cdot Casp \cdot (DSB_{\max} - DNA_{DSB})$		
$\boldsymbol{DNA_{DSB}} \rightarrow \varnothing$	$\frac{rep}{DNA_{DSB} + DSB_{\mathrm{rep}}}$	rep<br>DNA_{DSB}	10^{-3}/s<br>20
$\boldsymbol{ATM_{\mathrm{u}}} \xrightarrow{DSB} \boldsymbol{ATM_{\mathrm{p}}}$	$p_1 \cdot \frac{DNA_{DSB}^h}{M_1^h + DNA_{DSB}^h}$	p_1<br>h<br>M_1	3×10^{-4}/s<br>2<br>5
$\boldsymbol{ATM_{\mathrm{u}}} \xleftarrow{Wip1} \boldsymbol{ATM_{\mathrm{p}}}$	$d_1 \cdot Wip1$	d_1	10^{-8}/s
$\boldsymbol{SIAH\text{-}1_{\mathrm{u}}} \xrightarrow{ATM_{\mathrm{p}}} \boldsymbol{SIAH\text{-}1_{\mathrm{p}}}$	$p_2 \cdot ATM_{\mathrm{p}}$	p_2	10^{-8}/s
$\boldsymbol{SIAH\text{-}1_{\mathrm{u}}} \leftarrow \boldsymbol{SIAH\text{-}1_{\mathrm{p}}}$	d_2	d_2	3×10^{-5}/s
$\boldsymbol{\varnothing \rightarrow HIPK2}$	s_8	s_8	30/s
$\boldsymbol{HIPK2} \xrightarrow{Mdm2_{\mathrm{nuc\_2p}},\ SIAH\text{-}1_{\mathrm{u}}} \varnothing$	$g_7 \cdot (SIAH\text{-}1_{\mathrm{u}} + Mdm2_{\mathrm{nuc\_2p}})^2$	g_7	10^{-13}/s
$\boldsymbol{Wip1_{gene\_i}} \rightarrow \boldsymbol{Wip1_{gene\_a}}$	q_{0_Wip1}	q_{0_Wip1}	10^{-5}/s
$\boldsymbol{Wip1_{gene\_i}} \xrightarrow{p53_{\mathrm{ARRESTER}}} \boldsymbol{Wip1_{gene\_a}}$	$q_{1\_\mathrm{Wip1}} \cdot p53_{\mathrm{ARRESTER}}^h$	q_{1_Wip1}<br>h	3×10^{-13}/s<br>2
$\boldsymbol{Wip1_{gene\_i}} \leftarrow \boldsymbol{Wip1_{gene\_a}}$	q_2	q_2	3×10^{-3}/s
$\varnothing \xrightarrow{Wip1_{gene\_a}} \boldsymbol{Wip1_{\mathrm{mRNA}}}$	$s_1 \cdot Wip1_{gene\_a}$	s_1	0.1/s
$\boldsymbol{Wip1_{\mathrm{mRNA}}} \rightarrow \varnothing$	g_1	g_1	3×10^{-4}/s
$\varnothing \rightarrow \boldsymbol{Wip1}$	$t_1 \cdot Wip1_{\mathrm{mRNA}}$	t_1	0.1/s
$\boldsymbol{Wip1} \rightarrow \varnothing$	g_8	g_8	3×10^{-4}/s
$\varnothing \rightarrow \boldsymbol{p53_{0\mathrm{p}}}$	s_6	s_6	300/s
$\boldsymbol{p53_{0\mathrm{p}}} \rightarrow \varnothing$	g_{101}	g_{101}	10^{-5}/s
$\boldsymbol{p53_{\mathrm{s46}}} \rightarrow \varnothing$			
$\boldsymbol{p53_{\mathrm{ARRESTER}}} \rightarrow \varnothing$			
$\boldsymbol{p53_{\mathrm{KILLER}}} \rightarrow \varnothing$			
$\boldsymbol{p53_{0\mathrm{p}}} \xrightarrow{Mdm2_{\mathrm{nuc\_2p}}} \varnothing$	$g_{11} \cdot Mdm2_{\mathrm{nuc\_2p}}^2$	g_{11}	10^{-11}/s
$\boldsymbol{p53_{\mathrm{ARRESTER}}} \xrightarrow{Mdm2_{\mathrm{nuc\_2p}}} \varnothing$	$g_{12} \cdot Mdm2_{\mathrm{nuc\_2p}}^2$	g_{12}	10^{-13}/s
$\boldsymbol{p53_{\mathrm{KILLER}}} \xrightarrow{Mdm2_{\mathrm{nuc\_2p}}} \varnothing$			
$\boldsymbol{p53_{\mathrm{s46}}} \xrightarrow{Mdm2_{\mathrm{nuc\_2p}}} \varnothing$			
$\mathbf{p53_{0p}} \xrightarrow{ATM_{\mathrm{p}}} \boldsymbol{p53_{\mathrm{ARRESTER}}}$	$p_3 \cdot ATM_{\mathrm{p}}$	p_3	3×10^{-8}/s
$\boldsymbol{p53_{\mathrm{s46}}} \xrightarrow{ATM_{\mathrm{p}}} \boldsymbol{p53_{\mathrm{KILLER}}}$			
$\boldsymbol{p53_{0\mathrm{p}}} \leftarrow \boldsymbol{p53_{\mathrm{ARRESTER}}}$	d_3	d_3	10^{-4}/s
$\boldsymbol{p53_{\mathrm{s46}}} \leftarrow \boldsymbol{p53_{\mathrm{KILLER}}}$			

$p53_{0p} \xrightarrow{HIPK2} p53_{s46}$ $p53_{ARRESTER} \xrightarrow{HIPK2} p53_{KILLER}$	$p_4 \cdot HIPK2$	p_4	10^{-10}/s
$p53_{0p} \xleftarrow{Wip1} p53_{s46}$ $p53_{ARRESTER} \xleftarrow{Wip1} p53_{KILLER}$	$d_4 \cdot Wip1$	d_4	10^{-10}/s
$Mdm2_{gene\_i} \rightarrow Mdm2_{gene\_a}$	q_{0_Mdm2}	q_{0_Mdm2}	10^{-4}/s
$Mdm2_{gene\_i} \xrightarrow{p53_{ARRESTER}} Mdm2_{gene\_a}$	$q_{1\_Mdm2} \cdot p53^{h}_{ARRESTER}$	q_{1_Mdm2} h	3×10^{-13}/s 2
$Mdm2_{gene\_i} \leftarrow Mdm2_{gene\_a}$	q_2	q_2	3×10^{-3}/s
$\emptyset \xrightarrow{Mdm2_{gene\_a}} Mdm2_{mRNA}$	$s_3 \cdot Mdm2_{gene\_a}$	s_3	0.1/s
$Mdm2_{mRNA} \rightarrow \emptyset$	g_3	g_3	3×10^{-4}/s
$\emptyset \rightarrow Mdm2_{cyt\_0p}$	$t_3 \cdot Mdm2_{mRNA}$	t_3	0.1/s
$Mdm2_{cyt\_0p} \xrightarrow{AKT_p} Mdm2_{cyt\_2p}$	$p_5 \cdot Akt_p$	p_5	10^{-8}/s
$Mdm2_{cyt\_0p} \leftarrow Mdm2_{cyt\_2p}$	d_5	d_5	10^{-4}/s
$Mdm2_{cyt\_2p} \rightarrow Mdm2_{nuc\_2p}$	i_1	i_1	10^{-3}/s
$Mdm2_{nuc\_2p} \xrightarrow{ATM_p} Mdm2_{nuc\_3p}$	$p_6 \cdot ATM_p$	p_6	10^{-8}/s
$Mdm2_{nuc\_2p} \xleftarrow{Wip1} Mdm2_{nuc\_3p}$	$d_6 \cdot Wip1$	d_6	10^{-10}/s
$Mdm2_{cyt\_0p} \rightarrow \emptyset$	g_{14}	g_{14}	10^{-4}/s
$Mdm2_{cyt\_2p} \rightarrow \emptyset$ $Mdm2_{nuc\_2p} \rightarrow \emptyset$	g_{15}	g_{15}	3×10^{-5}/s
$Mdm2_{nuc\_3p} \rightarrow \emptyset$	g_{16}	g_{16}	10^{-4}/s
$PTEN_{gene\_i} \rightarrow PTEN_{gene\_a}$	q_{0_PTEN}	q_{0_PTEN}	10^{-5}/s
$PTEN_{gene\_i} \xrightarrow{p53_{KILLER}} PTEN_{gene\_a}$	$q_{1\_PTEN} \cdot p53^{h}_{KILLER}$	q_{1_PTEN} h	3×10^{-13}/s 2
$PTEN_{gene\_i} \leftarrow PTEN_{gene\_a}$	q_2	q_2	3×10^{-3}/s
$\emptyset \xrightarrow{PTEN_{gene\_a}} PTEN_{mRNA}$	$s_2 \cdot PTEN_{gene\_a}$	s_2	0.03/s
$PTEN_{mRNA} \rightarrow \emptyset$	g_2	g_2	3×10^{-4}/s
$\emptyset \rightarrow PTEN$	$t_2 \cdot PTEN_{mRNA}$	t_2	0.1/s
$PTEN \rightarrow \emptyset$	g_6	g_6	3×10^{-5}/s
$PIP2 \xrightarrow{PI3K} PIP3$	$p_8 \cdot PI3K$	p_8	3×10^{-9}/s
$PIP2 \xleftarrow{PTEN} PIP3$	$d_7 \cdot PTEN$	d_7	3×10^{-7}/s
$Akt_u \xrightarrow{PIP3} Akt_p$	$p_{12} \cdot PIP3$	p_{12}	10^{-9}/s
$Akt_u \leftarrow Akt_p$	d_8	d_8	10^{-4}
**Apoptotic module**			
$Bax_{gene\_i} \rightarrow Bax_{gene\_a}$	q_{0_Bax}	q_{0_Bax}	10^{-5}/s

$\boldsymbol{Bax_{gene\_i}} \xrightarrow{\boldsymbol{p53_{\text{KILLER}}}} \boldsymbol{Bax_{gene\_a}}$	$q_{1\_Bax} \cdot p53^{h}_{\text{KILLER}}$	q_{1_Bax}<br>h	3×10^{-13}/s<br>2
$\boldsymbol{Bax_{gene\_i}} \leftarrow \boldsymbol{Bax_{gene\_a}}$	q_2	q_2	3×10^{-3}/s
$\varnothing \xrightarrow{\boldsymbol{Bax_{gene\_a}}} \boldsymbol{Bax_{\text{mRNA}}}$	$s_4 \cdot Bax_{gene\_a}$	s_4	0.03/s
$\boldsymbol{Bax_{\text{mRNA}}} \rightarrow \varnothing$	g_4	g_4	3×10^{-4}/s
$\varnothing \rightarrow \boldsymbol{Bax}$	$t_4 \cdot Bax_{\text{mRNA}}$	t_4	0.1/s
$\boldsymbol{Bax} \rightarrow \varnothing$	g_9	g_9	10^{-4}/s
$\boldsymbol{Bax} + \boldsymbol{Bclx_{\text{L}}} \rightarrow \boldsymbol{Bax}{:}\,\boldsymbol{Bclx_{\text{L}}}$	b_1	b_1	3×10^{-5}/s
$\boldsymbol{Bax} + \boldsymbol{Bclx_{\text{L}}} \leftarrow \boldsymbol{Bax}{:}\,\boldsymbol{Bclx_{\text{L}}}$	u_1	u_1	10^{-3}/s
$\boldsymbol{Bax}{:}\,\boldsymbol{Bclx_{\text{L}}} \rightarrow \boldsymbol{Bclx_{\text{L}}}$	g_{16}	g_{16}	10^{-4}/s
$\boldsymbol{Bclx_{\text{L}}} + \boldsymbol{Bad_{\text{u}}} \rightarrow \boldsymbol{Bclx_{\text{L}}}{:}\,\boldsymbol{Bad_{\text{u}}}$	b_2	b_2	3×10^{-3}/s
$\boldsymbol{Bclx_{\text{L}}} + \boldsymbol{Bad_{\text{u}}} \leftarrow \boldsymbol{Bclx_{\text{L}}}{:}\,\boldsymbol{Bad_{\text{u}}}$	u_2	u_2	10^{-3}/s
$\boldsymbol{Bclx_{\text{L}}}{:}\,\boldsymbol{Bad_{\text{u}}} \xrightarrow{\boldsymbol{Akt_{\text{p}}}} \boldsymbol{Bclx_{\text{L}}}$	$p_7 \cdot Akt_{\text{p}}$	p_7	3×10^{-9}/s
$\boldsymbol{Bad_{\text{u}}} \xrightarrow{\boldsymbol{AKT_{\text{p}}}} \boldsymbol{Bad_{\text{p}}}$	$p_7 \cdot Akt_{\text{p}}$	p_7	3×10^{-9}/s
$\boldsymbol{Bad_{\text{u}}} \leftarrow \boldsymbol{Bad_{\text{p}}}$	d_9	d_9	3×10^{-5}/s
$\boldsymbol{Bad_{\text{u}}}$ + **14-3-3** $\leftarrow \boldsymbol{Bad_{\text{p}}}$ + **14-3-3**			
$\boldsymbol{Bad_{\text{p}}}$ + **14-3-3** $\rightarrow \boldsymbol{Bad_{\text{p}}}$: **14-3-3**	b_3	b_3	3×10^{-3}/s
$\boldsymbol{Bad_{\text{p}}}$ + **14-3-3** $\leftarrow \boldsymbol{Bad_{\text{p}}}$: **14-3-3**	u_3	u_3	10^{-3}/s
$\varnothing \rightarrow \boldsymbol{proCasp}$	s_7	s_7	30/s
$\boldsymbol{proCasp} \xrightarrow{\boldsymbol{Bax,\ Casp}} \boldsymbol{Casp}$	$a_1 \cdot Bax + a_2 \cdot Casp^2$	a_1<br>a_2	3×10^{-10}/s<br>10^{-12}/s
$\boldsymbol{proCasp} \rightarrow \varnothing$	g_{17}	g_{17}	3×10^{-4}/s
$\boldsymbol{Casp} \rightarrow \varnothing$			
**Cell cycle arrest module**			
$\boldsymbol{p21_{gene\_i}} \rightarrow \boldsymbol{p21_{gene\_a}}$	q_{0_p21}	q_{0_p21}	10^{-5}/s
$\boldsymbol{p21_{gene\_i}} \xrightarrow{\boldsymbol{p53_{\text{ARRESTER}}}} \boldsymbol{p21_{gene\_a}}$	$q_{1\_p21} \cdot p53^{h}_{\text{ARRESTER}}$	q_{1_p21}<br>h	3×10^{-13}/s<br>2
$\boldsymbol{p21_{gene\_i}} \leftarrow \boldsymbol{p21_{gene\_a}}$	q_2	q_2	3×10^{-3}/s
$\varnothing \xrightarrow{\boldsymbol{p21_{gene\_a}}} \boldsymbol{p21_{\text{mRNA}}}$	$s_5 \cdot p21_{gene\_a}$	s_5	0.1/s
$\boldsymbol{p21_{\text{mRNA}}} \rightarrow \varnothing$	g_5	g_5	3×10^{-4}/s
$\varnothing \rightarrow \boldsymbol{p21}$	$t_5 \cdot p21_{\text{mRNA}}$	t_5	0.1/s
$\boldsymbol{p21} \rightarrow \varnothing$	g_{19}	g_{19}	3×10^{-4}/s
$\varnothing \xrightarrow{\boldsymbol{E2F1}} \boldsymbol{CycE}$	$s_9 \cdot \dfrac{E2F1^h}{{M_3}^h + E2F1^h}$	s_9<br>h<br>M_3	30/s<br>2<br>2×10^5
$\varnothing \rightarrow \boldsymbol{CycE}$	s_{10}	s_{10}	3/s
$\boldsymbol{p21} + \boldsymbol{CycE} \rightarrow \boldsymbol{p21}{:}\,\boldsymbol{CycE}$	b_5	b_5	10^{-5}/s
$\boldsymbol{p21} + \boldsymbol{CycE} \leftarrow \boldsymbol{p21}{:}\,\boldsymbol{CycE}$	u_6	u_6	10^{-4}/s
$\boldsymbol{CycE} \rightarrow \varnothing$	g_{20}	g_{20}	10^{-4}/s

$\boldsymbol{Rb1_u} \xrightarrow{\boldsymbol{CycE}} \mathbf{Rb1_p}$	$p_9 \cdot CycE$	p_9	3×10^{-6}/s
$\boldsymbol{Rb1_u} \leftarrow \boldsymbol{Rb1_p}$	$\frac{d_{12}}{M_2 + Rb1_\mathrm{p}}$	d_{12}<br>M_2	10^4/s<br>10^5
$\boldsymbol{Rb1_u + E2F1 \rightarrow Rb1_u : E2F1}$	b_4	b_4	10^{-5}/s
$\boldsymbol{Rb1_u + E2F1 \leftarrow Rb1_u : E2F1}$	u_5	u_5	10^{-4}/s
$\boldsymbol{Rb1_u : E2F1} \xrightarrow{\boldsymbol{CycE}} \boldsymbol{Rb1_p + E2F1}$	$p_{10} \cdot CycE$	p_{10}	3×10^{-6}/s

Supplementary references

S1. Hat B, Kochańczyk M, Bogdał MN, Lipniacki T. Feedbacks, Bifurcations, and Cell Fate Decision-Making in the p53 System. Meier-Schellersheim M, editor. PLoS Comput Biol. 2016;12: e1004787. doi:10.1371/journal.pcbi.1004787

S2. Zhang F, Tagen M, Throm S, Mallari J, Miller L, Guy RK, et al. Whole-Body Physiologically Based Pharmacokinetic Model for Nutlin-3a in Mice after Intravenous and Oral Administration. Drug Metab Dispos. 2011;39: 15–21. doi:10.1124/dmd.110.035915
